# MitoFLUX-EM reveals damaged mitochondria are captured by pre-existing mitolysosomes during PINK1-dependent mitophagy in the heart

**DOI:** 10.21203/rs.3.rs-11310455/v1

**Published:** 2026-10-20

**Authors:** Derek Narendra, Jennifer Petersen, Xiaoping Huang, Christelle Guillermier, Melissa Wilson, Damian Di Florio, Hsin-Pin Lin, Theresa D'Silva, Yan Li, Wolfdieter Springer

## Abstract

Damaged mitochondria are cleared by PINK1-Parkin mitophagy, but the pathway is incompletely understood in vivo. Here, we developed MitoFLUX-EM, combining electron microscopy with nanoscale ¹⁵N isotope tracing to resolve the temporal sequence of mitophagy intermediates in vivo, and applied it to a cardiomyopathy mouse model (CHCHD10 S59L) with high PINK1-dependent mitophagy. Unexpectedly, we found that damaged mitochondria are directly captured by pre-existing mitolysosomes, resulting in multisegmented mitolysosomes that sequestered as many as 33 mitochondria at distinct stages of ultrastructural degradation. Autophagosomal-like membranes arising from the mitolysosomes preferentially engaged the intact outer membrane of damaged mitochondria and neither protein removal nor rupture of the outer membrane was required for mitophagy, contrary to prior models. Pink1 loss disrupted this pathway, causing accumulation of aggregates within mitochondria and reduced survival. These findings reveal that PINK1-dependent mitophagy proceeds through a novel form of microautophagy to safeguard cardiac mitochondrial quality in vivo and establish MitoFLUX-EM as a platform for resolving organelle turnover.

